# Supplementary material for: Clusters of acidic and hydrophobic residues can predict acidic transcriptional activation domains from protein sequence
Source: Genetics. 2023 Jul 18;225(2):iyad131. doi: 10.1093/genetics/iyad131 (PMC10550315; doi:10.1093/genetics/iyad131)
Supplement: iyad131_Supplementary_Data [file iyad131_supplementary_data.zip › Supplemental Table Legends for Kotha and Staller 2023.docx]

Supplemental Table Legends for Kotha and Staller 2023

**Supplementary Data Tables**

Table S1: The gold standard list of activation domains.

Table S2: Removing each of the original eight AA from the predictor did not improve performance.

Table S3: Varying the length of the tiles did not improve performance.

Table S4: Using single amino acids as the y-axis of the predictor.

Table S5: Using pairs of amino acids as the y-axis of the predictor. The top 20 pairs are reported.

Table S6: Using triplets of amino acids as the y-axis of the predictor. The top 20 triplets are reported.

Table S7: Activation domains predicted by both our original predictor and the yeast neural networks.

Table S8: The coordinates and sequences of the activation domains predicted by the revised predictor.

Table S9: The coordinates and sequences of a consolidated list of published and experimentally identified activation domains. This table combines the gold standard list, the Soto list, the Staller et al 2018 list, and the DelRosso et al 2022 list.
