# Supplementary material for: Clusters of acidic and hydrophobic residues can predict acidic transcriptional activation domains from protein sequence
Source: Genetics. 2023 Jul 18;225(2):iyad131. doi: 10.1093/genetics/iyad131 (PMC10550315; doi:10.1093/genetics/iyad131)
Supplement: iyad131_Supplementary_Data [file iyad131_supplementary_data.zip › Supplemental_Figures_GENETICS-2023-306271.pdf]

Sanjana R. Kotha and Max Valentín Staller

Center for Computational Biology, University of California, Berkeley
Correspondence: mstaller@berkeley.edu

Supplemental Figures for:

Clusters of acidic and hydrophobic residues can predict acidic transcriptional activation domains from protein sequence

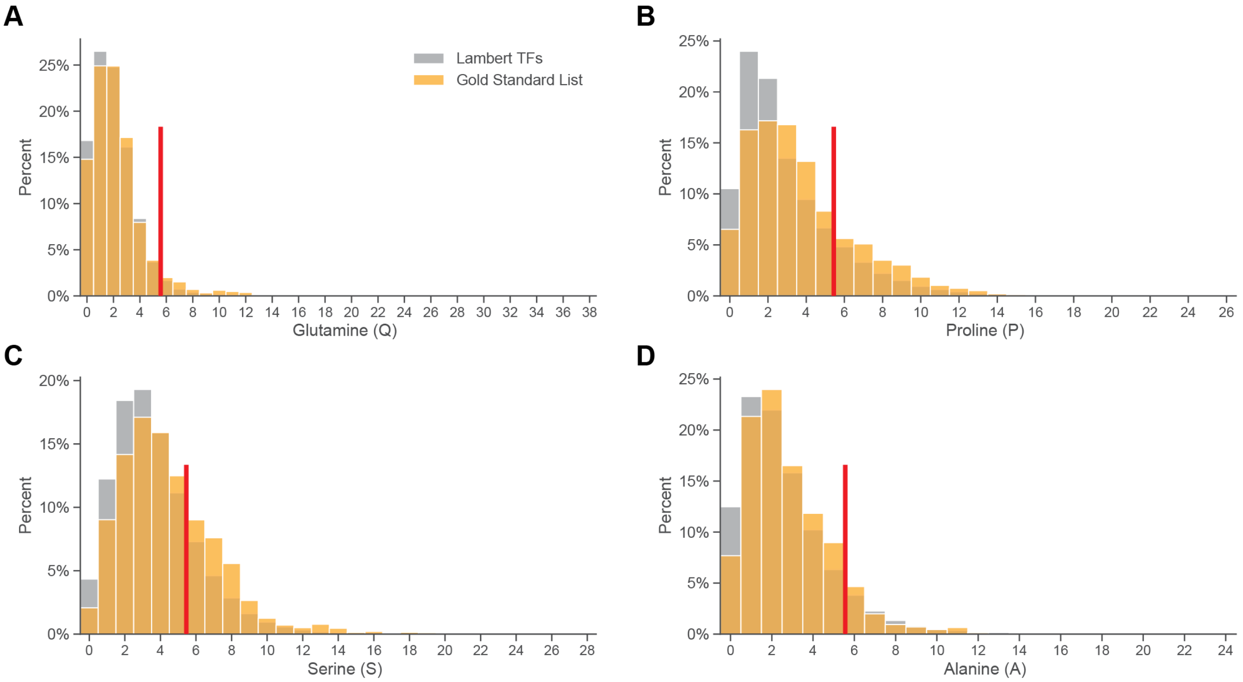

Figure S1: Histograms of additional sequence features of 39-AA tiles from activation domains on the gold standard list. The red lines indicate the threshold (15%) used to denote enrichment of specific amino acids, e.g. tiles with 6 or more prolines are proline-rich. The maximum value of the X-axis indicates the maximum value found, e.g. there exist tiles where 38/39 residues are glutamine. The tiles from the gold standard list of activation domains (orange) show a modest enrichment of glutamine, proline, and serine compared to tiles from all transcription factors.

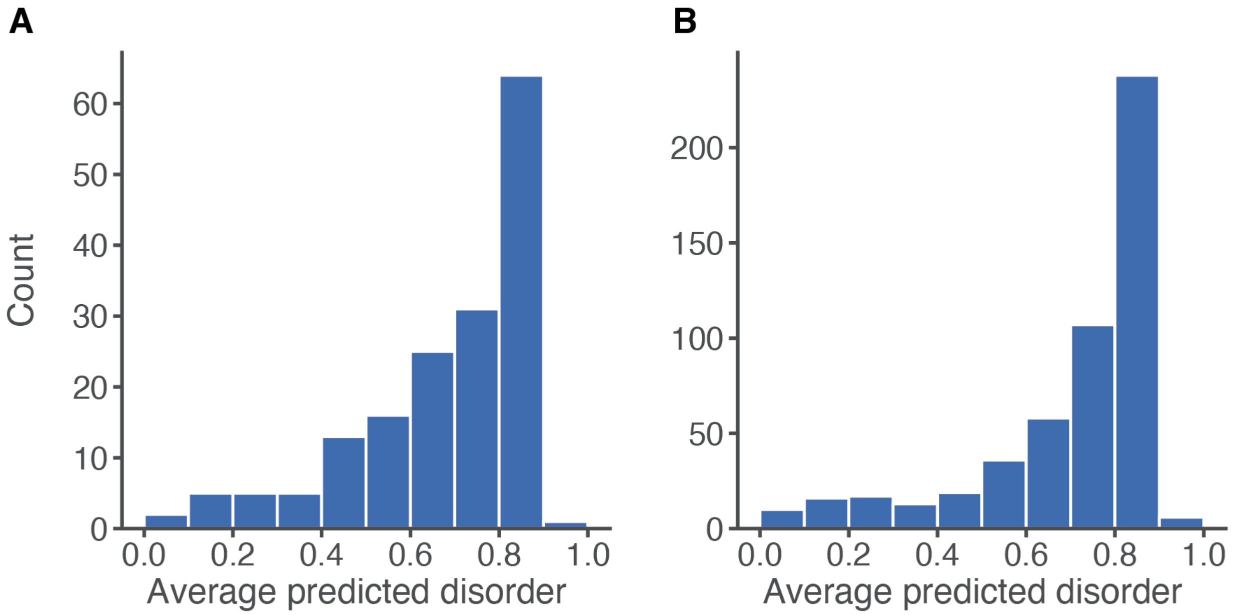

Figure S2: The majority of activation domains from the gold standard list (A) and the Soto list (B) are predicted to be intrinsically disordered with Metapredict2, which has been optimized to avoid common artifacts when predicting the disorder of transcription factor sequences. (Emenecker *et al.* 2022). For each full length activation domain, we computed the Metapredict2 score for each residue and then averaged it over all residues. Regions with a score above 0.5 are predicted to be disordered.

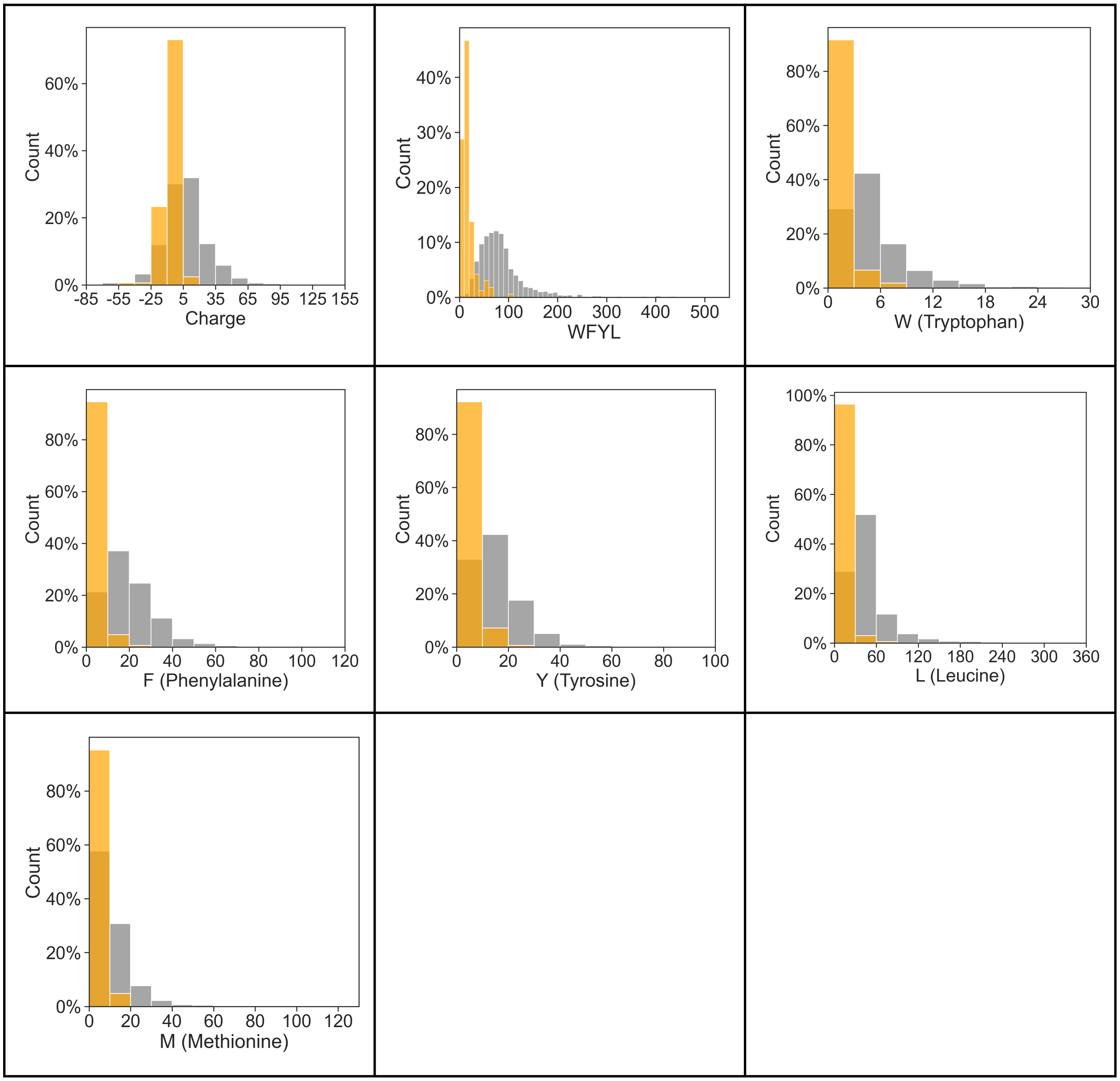

Figure S3: Histograms of amino acid frequencies in gold standard list activation domains before normalizing for length or splitting into tiles. Because all the activation domains had different lengths, these histograms are not particularly informative. This problem prompted us to decompose regions into 39-AA tiles. Gray, all transcription factors. Orange, gold standard list activation domains.

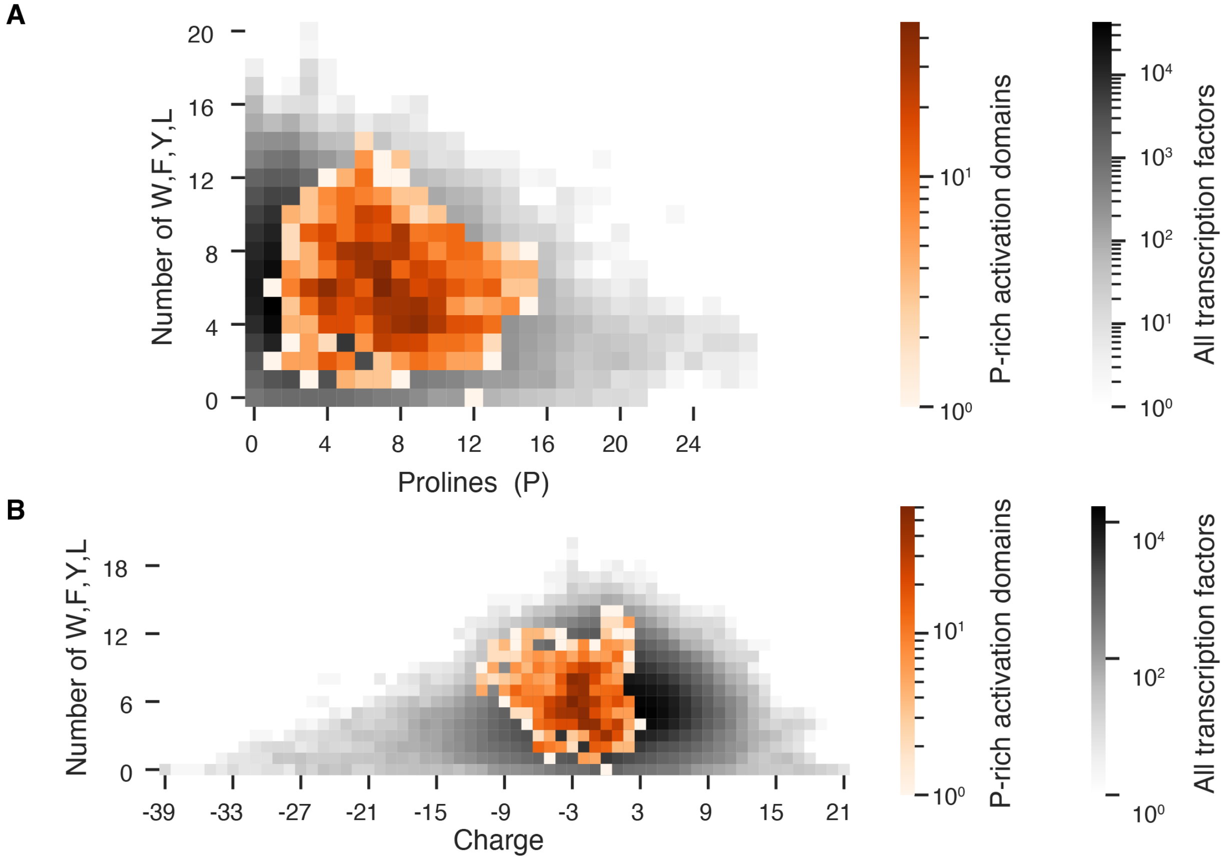

Figure S4: Proline-rich activation domains (orange heatmap) from the gold standard list are not among the most proline-rich regions of transcription factors (gray heatmap). For all 881K tiles from transcription factors, we counted P residues and WFYL residues. The combination of P and WFYL residues does not enrich P-rich activation domains. The tiles from P-rich activation domains often have net negative charges, but are less negative than acidic activation domains.

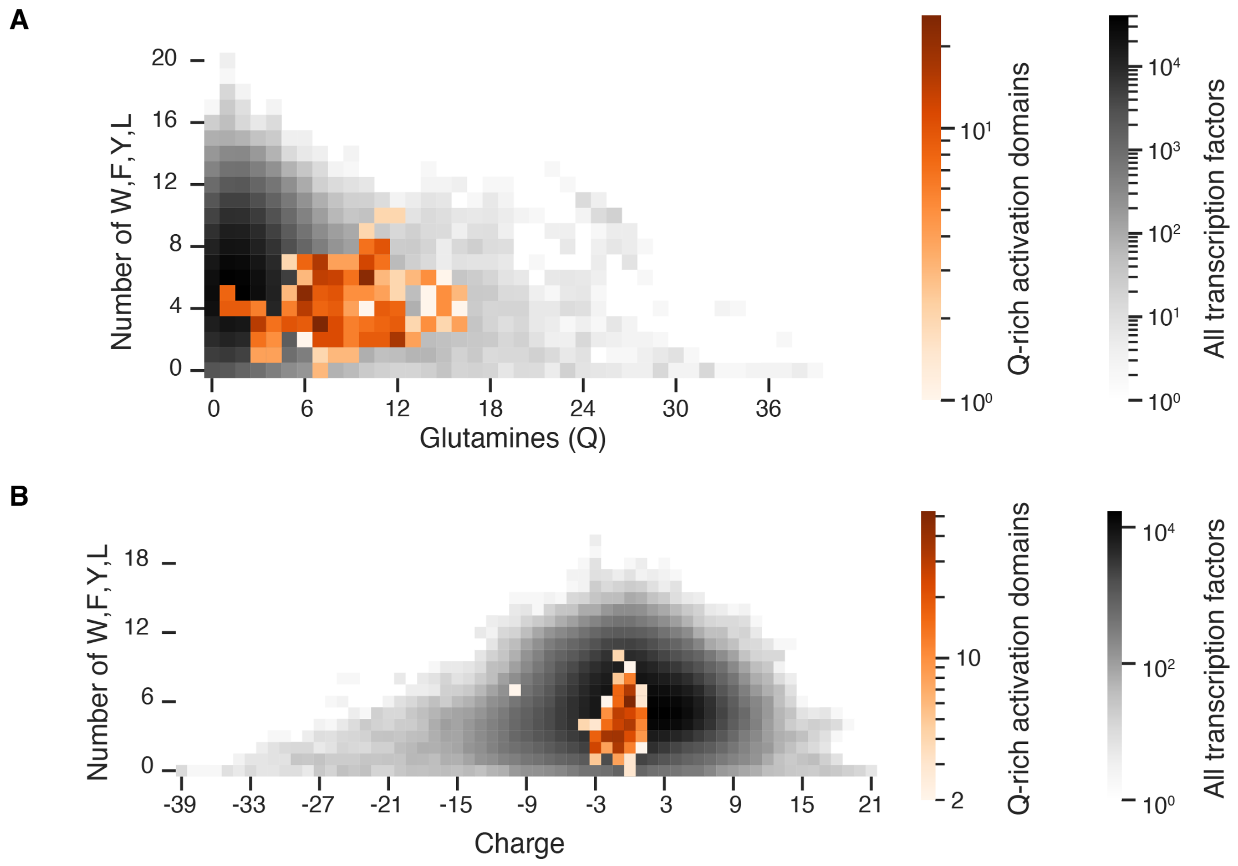

Figure S5: Glutamine-rich activation domains from the gold standard list are not among the most glutamine-rich regions of transcription factors. A) For all 881K tiles from transcription factors, we counted glutamine (Q) residues and WFYL residues. The combination of Q and WFYL residues does not enrich Q-rich activation domains. B) Most tiles from Q-rich activation domains are near neutral or slightly acidic.

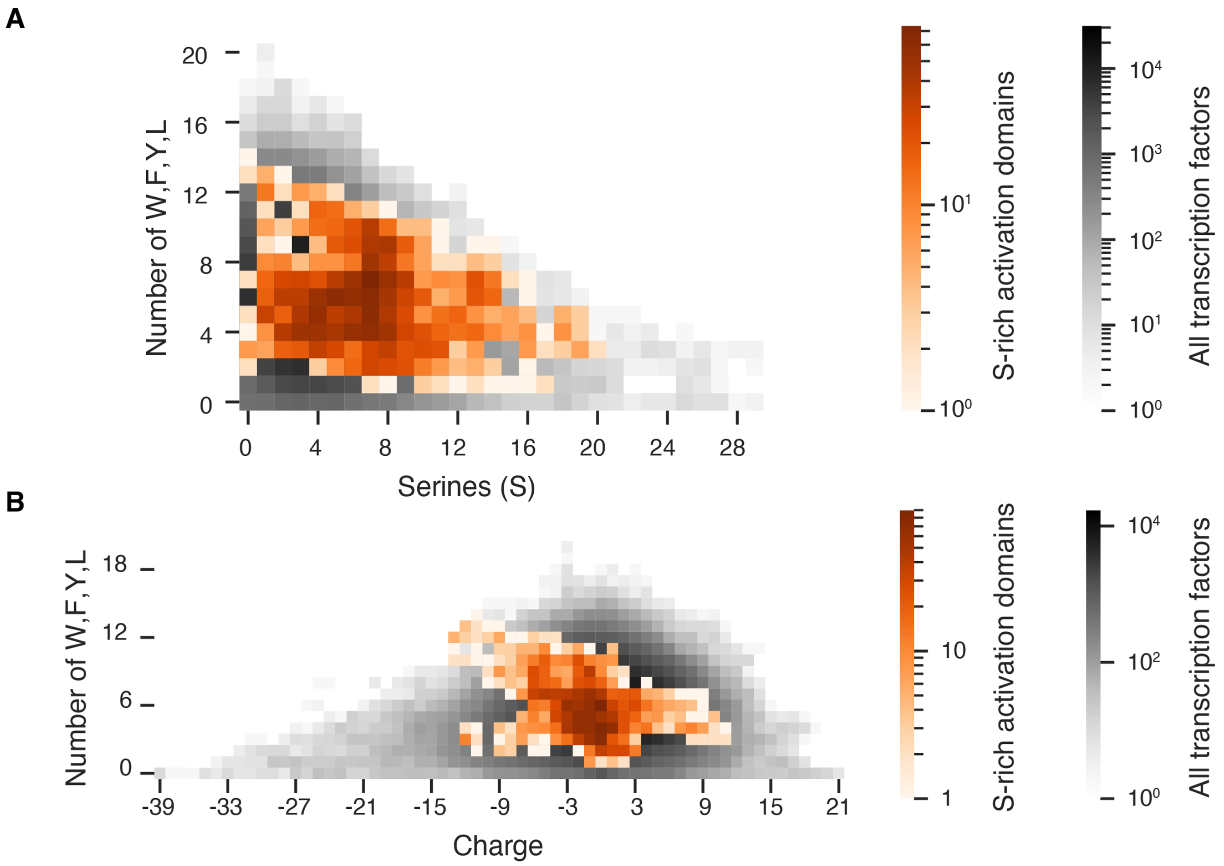

Figures S6: Serine-rich (S-rich) activation domains from the gold standard list are not among the most serine-rich regions of transcription factors. For all 881K tiles from transcription factors, we counted serine (S) residues and WFYL residues. The combination of S and WFYL residues does not enrich S-rich activation domains. The tiles from S-rich activation domains show a wide range of net charges, with some being very acidic.

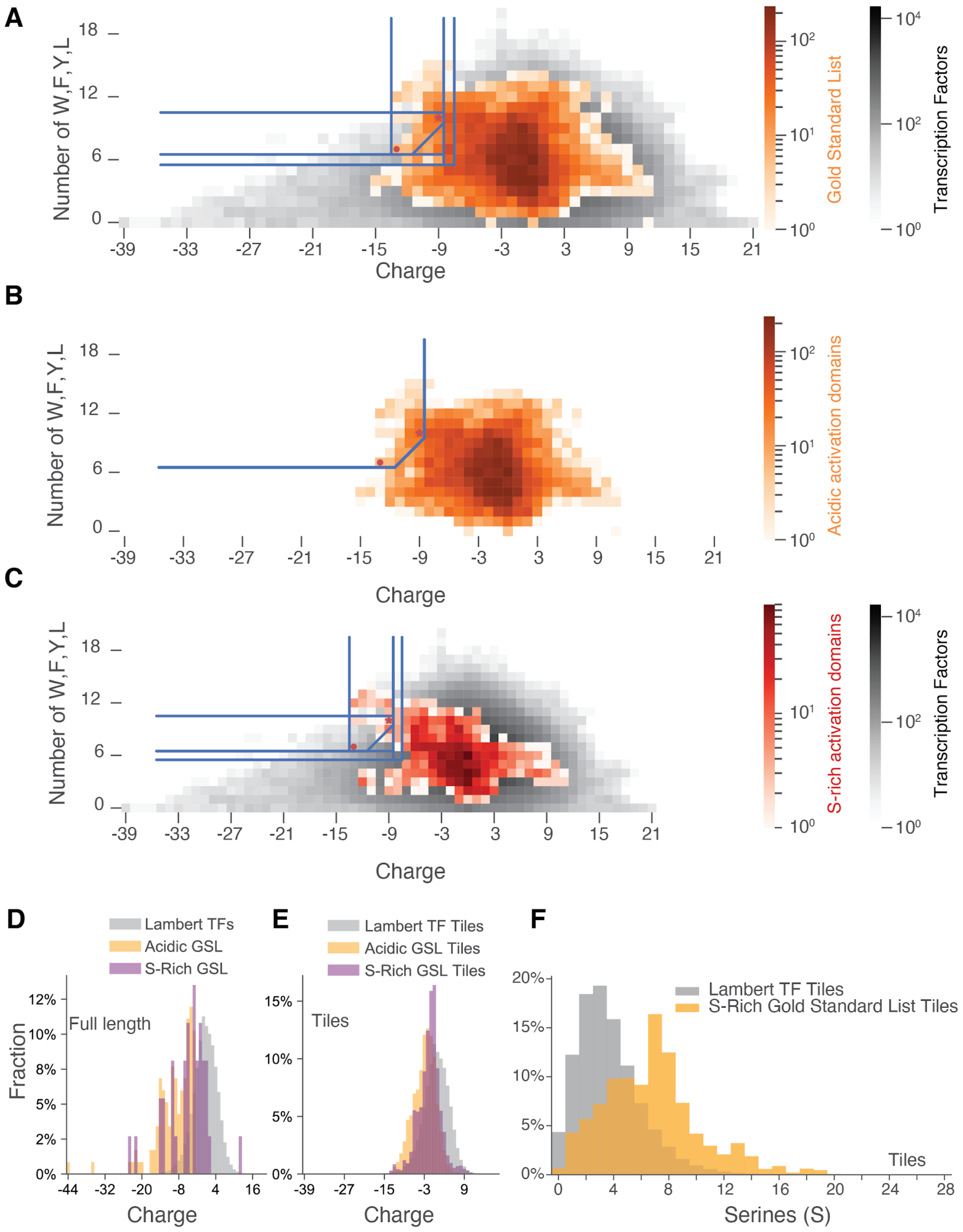

Figure S7: The distribution of sequence features in gold standard list acidic activation domains is the distribution of properties of all activation domains on the gold standard list.
A) Tiles from all activation domains from the gold standard list (n = 167).

B) Tiles from the acidic activation domains on the gold standard list (n = 105). There are fewer positively charged tiles.
C) Tiles from the S-rich activation domains on the gold standard list (n = 37). Although some S-rich activation domains are also acidic, the acidic activation domain predictor struggles to predict the majority of S-rich activation domains. S-rich activation domains are determined by a different mixture of sequence features.
D) Histograms of the net charge of full length regions, comparing transcription factors, acidic activation domains from the gold standard list (GSL), and S-rich activation domains. The acidic activation domains have the most acidity, but some S-rich activation domains are very negatively charged.
E) Similar to D, but using the tiles from each set. In general, S-rich activation domains are more acidic than transcription factors. Acidic activation domains are the most negatively charged class. There is some overlap between acidic activation domains and S-rich activation domains.
F) Tiles from S-rich activation domains contain more serine residues than tiles from all transcription factors, as expected.

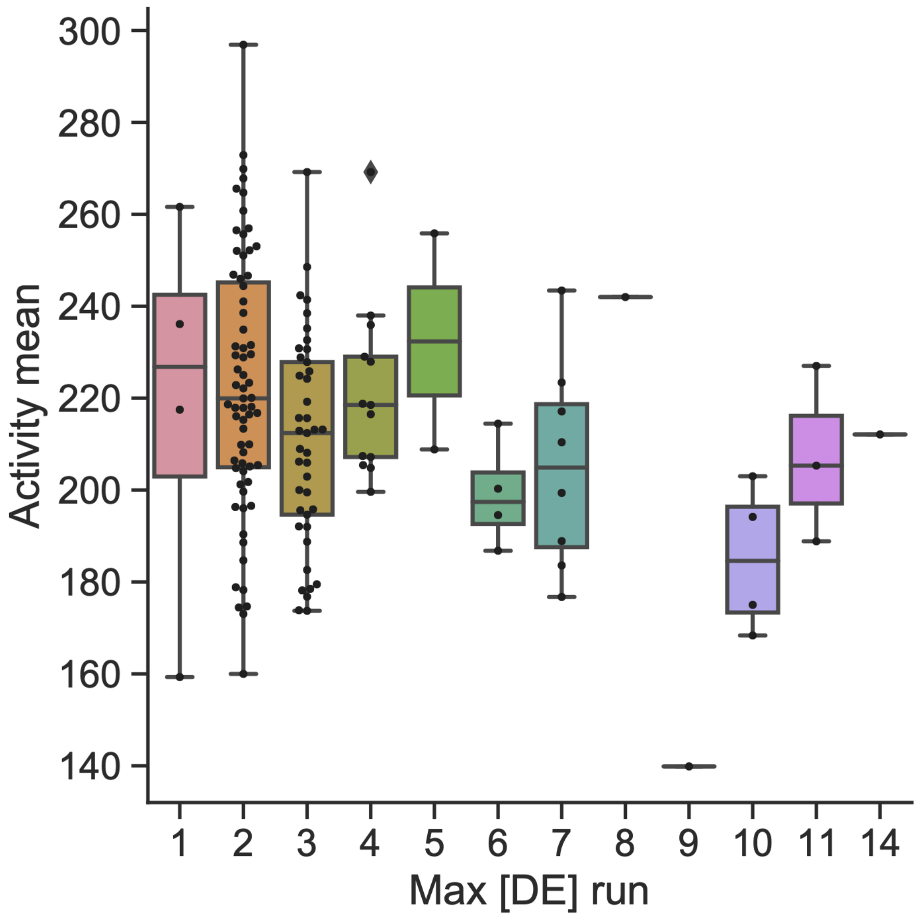

Figure S8: Long runs of acidic residues are depleted from strong activation domains. These are the tested predictions from the original predictor, replotting published data (Staller *et al.* 2022). For each activation domain, we counted the longest contiguous run of acidic residues (D or E) and plotted it against activity. In this experiment, the no activation domain control was normalized to 200. Strong activation domains had activity greater than 221. Boxplots show median and interquartile range, whiskers show full range of the data. Sequences with long runs of acidic residues tended to have lower activity, but the effect is weak.

References

Emenecker R. J., D. Griffith, and A. S. Holehouse, 2022 Metapredict V2: An update to metapredict, a fast, accurate, and easy-to-use predictor of consensus disorder and structure. bioRxiv 2022.06.06.494887.

Staller M. V., E. Ramirez, S. R. Kotha, A. S. Holehouse, R. V. Pappu, *et al.*, 2022 Directed mutational scanning reveals a balance between acidic and hydrophobic residues in strong human activation domains. Cell Syst 13: 334–345.e5.
